# Patients with worsening chronic heart failure who present to a hospital emergency department require hospital care

**DOI:** 10.1186/1756-0500-5-132

**Published:** 2012-03-08

**Authors:** Masoud Shafazand, Harshidaben Patel, Inger Ekman, Karl Swedberg, Maria Schaufelberger

**Affiliations:** 1 Department of Emergency and Cardiovascular Medicine, Sahlgrenska Academy, Sahlgrenska University Hospital/Östra, University of Gothenburg, Gothenburg, Sweden; 2 Institute of Health and Care Sciences, Sahlgrenska Academy at University of Gothenburg, Gothenburg, Sweden. The Regional Ethical Review Board: The Sahlgrenska Academy, Ethics committee

**Keywords:** Chronic heart failure, Hospitalisation, Deterioration, Emergency care

## Abstract

**Background:**

Chronic heart failure (CHF) is a major public health problem characterised by progressive deterioration with disabling symptoms and frequent hospital admissions. To influence hospitalisation rates it is crucial to identify precipitating factors.

To characterise patients with CHF who seek an emergency department (ED) because of worsening symptoms and signs and to explore the reasons why they are admitted to hospital.

**Method:**

Patients (n = 2,648) seeking care for dyspnoea were identified at the ED, Sahlgrenska University Hospital/Östra. Out of 2,648 patients, 1,127 had a previous diagnosis of CHF, and of these, 786 were included in the present study with at least one sign and one symptom of worsening CHF.

**Results:**

Although several of the patients wanted to go home after acute treatment in the ED, only 2% could be sent home. These patients were enrolled in an interventional study, which evaluated the acute care at home compared to the conventional, in hospital care. The remaining patients were admitted to hospital because of serious condition, including pneumonia/respiratory disease, myocardial infarction, pulmonary oedema, anaemia, the need to monitor cardiac rhythm, pathological blood chemistry and difficulties to communicate.

**Conclusion:**

The vast majority of patients with worsening CHF seeking the ED required hospital care, predominantly because of co-morbidities. Patients with CHF with symptomatic deterioration may be admitted to hospital without additional emergency room investigations.

## Background

Chronic heart failure (CHF) is a major public health problem and an increasing burden on health care providers and society [1]. It is commonly associated with other chronic conditions e.g. coronary heart disease (CHD), chronic obstructive pulmonary disease (COPD), cardiacarrhythmia and diabetes [2]. CHF is often characterised by progressive deterioration with disabling symptoms and frequent hospital admissions [3]. The annual cost for treatment of CHF in Sweden is about 2% of the Swedish health care budget, with the major part (75%) constituting hospital care [4]. As described in the Swedish national guidelines, the majority of patients with heart failure are managed by primary care physicians [5]. Hospitalisation is common in these patients and in a recent study it was shown that 31% of the patients with heart failure (HF) in primary care were hospitalised at least once during follow-up over a 2- year period [6]. Conversely, 65% of patients hospitalised for HF are followed over an extended period of time in primary care [7]. Patients who develop acute HF or present with severe symptoms are admitted directly to hospital for treatment without first visiting a primary care physician [5]. In addition, many of the patients controlled in the hospital heart failure ambulatory department are sent to the emergency department (ED) if their condition deteriorates. While the number of hospital beds in Sweden has been reduced from 100,000 in the 1980s to 26 000 in 2005 during the past three decades, a reduction largely due to health care reforms, financial pressures (e.g., budget cuts) and rationalisation [8], a hospital readmission rate of 30-50% within 6 months after discharge has been reported for patients with CHF [3]. Increases in the prevalence of HF hospitalizations have also been reported from other countries, e.g. Scotland [9], the Netherlands [10], Spain [11], Singapore [12], Hong Kong [13], and USA [14]. Overall, survival in CHF is poor, where the 5-year survival rate in patients diagnosed in the late 1980s was about 40% [15]. However, from the late 1980s and coinciding with the introduction of new treatments, such as angiotensin-converting enzyme (ACE) inhibitors [16], beta-blockers [17], angiotensin receptor blockers (ARB) [18] and aldosterone antagonists [19], long-term mortality for CHF has decreased dramatically during the past two decades.

Even so, motality remains high for patients with HF and continues to be a serious public health problem in developing countries [20]. Shortness of breath, fatigue and fluid retention are hallmarks of the CHF condition [21]. As many as 32 symptoms have been described, including dyspnoea, fatigue, pain, anxiety and loss of appetite, depression and sleeping disorders [22]. We have previously shown that dyspnoea is the most frequent symptom in patients with deteriorating CHF that seek emergency care [22]. There is no evidence that patients with milder forms of CHF are hospitalised more often today than in the past. Home-based care of patients with worsening CHF after initial discharge from hospital may reduce re-hospitalisation, mortality or both [23]. Many patients treated for CHF experience worsening symptoms long before seeking medical attention [22], which, if discovered earlier, might be managed at home rather than in hospital. Knowledge about factors related to re-hospitalisation in patients with CHF is important in designing measures to prevent deterioration and avoid hospital admission. A few studies that have investigated the pattern of hospital readmission in patients with worsening CHF identified abnormalities (e.g., respiratory infection, arrhythmia, non adherence to prescribed treatment, coronary ischaemia and inadequate preadmission treatment) associated with clinical deterioration before admission [24-26]. However, only one of these studies is from northern Europe and that one is more than 10 years old [24]. To influence hospitalisation rates of patients with worsening CHF it is crucial to identify the precipitating factors of this condition. The present study had two objectives. The first was to characterise patients who seek the ED because of worsening CHF symptoms or signs. The second objective was to investigate the reasons why patients with worsening CHF require hospital care.

## Methods

Between April 2004 and May 2006, patients seeking care for dyspnoea were identified at the ED at Sahlgrenska University Hospital/Östra, Göteborg, Sweden, a hospital covering approximately 250 000 inhabitants and with about 40 000 annual visits at the ED. The ED is divided into an internal medicine unit and a unit devoted to surgery care. Almost 18,000 visits per year are related to medical conditions and a little more than half of these patients (54%) are admitted to the department of medicine (15% of all these admissions were related to HF). The registration of patients and data collection was performed by emergency and study physicians and study nurses. All the patients visiting emergency department seeking care for dyspnoea were identified and were registered. From this population, patients with known CHF were registered and details recorded regarding gender, age, socio-economic status, heart rate, blood pressure, symptoms and signs related to CHF. Patients with CHF that were included because of their declining condition had a prior diagnosis of CHF based on the European Society of Cardiology (ESC) Guidelines [21]. In addition, these patients complained of dyspnoea and required hospital care according to the attending physician. All these patients were invited to participate in a study reported elsewhere [27] if they fulfilled all the inclusion criteria (Table 1). In the present study, which was retrospectively conducted, a number of demographic variables were registered and the reasons for admission as well as echocardiographic findings were retrieved from patient records. Approval was obtained from The Regional Ethical Review Board (The Sahlgrenska Academy, Ethics committee) and all participants gave their written informed consent. The study conforms to the principles outlined in the Declaration of Helsinki (international guidelines for medical research on human subjects).

**Table 1:** Criteria for inclusion in the registry of patients with a diagnosis of chronic heart failure (CHF) seeking emergency care

Inclusion criteria	Prior diagnosis of CHF with diastolic or systolic left ventricular dysfunction
	Deterioration of CHF ≥ 3 days with symptoms of increasing dyspnoea, orthopnoea, weight gain ≥ 2 kg, debuting peripheral oedema or abdominal swelling

	Signs of fluid retention or myocardial dysfunction, such as extended jugular vein, leg oedema, tachypnoea, pulmonary crackles, ascites and third heart sound

	At least one symptom and one sign should be present

	New York Heart Association class II - IV

**Systolic heart failure was defined as:**	Ejection fraction ≦ 45%.

**Heart failure with preserved ejection fraction was defined as:**	Ejection fraction > 45% and signs of diastolic dysfunction:

	One of the following criteria should be fulfilled:

	- Posterior wall thickness + interventricular septum thickness/2 > 1.3 cm.

	- Enlarged left atrium (female > 42 mm, male > 46 mm) in absence of atrial fibrillation.

### Statistical analysis

Statistical analyses were performed using SPSS version 14.0 for windows (SPSS Inc., Chicago, IL, USA). Descriptive statistics were used to describe frequencies, percentages, medians, means and standard deviations (SD). Continuous variables were compared using Student's *t *test. A nominal significance level of 0.05 was used (all tests were two-tailed).

## Results

**Figure 1:**
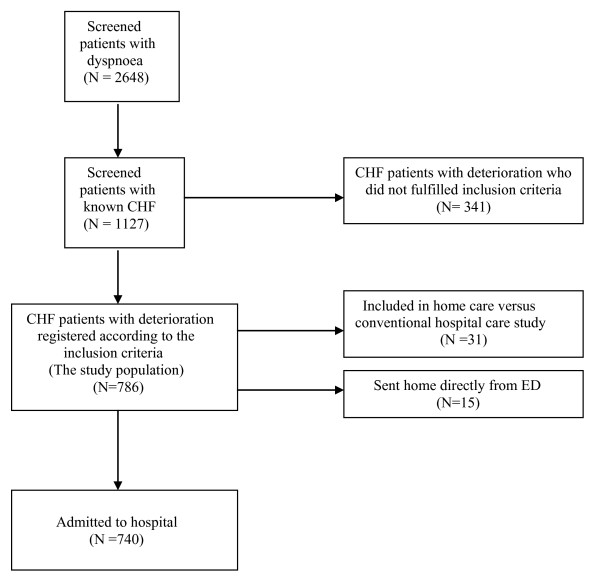
**Flow chart of patients and data availability**.

We screened 2,648 (51% women) patients, who sought emergency department because of dyspnoea during the study period (mean age 75 years; SD 14 years) (Figure 1). In the screened cohort of patients with dyspnoea, the mean age for women was 77 years (SD 15 years) and for the men was 73 years (SD 14 years) (p < 0.001). In the screened cohort of patients with dyspnoea, 1,127 (46% women) were previously diagnosed with CHF (mean age 79 years; SD 11 years). The mean age of those patients with CHF was for women 82 years (SD 12 years) years and for men 76 years (SD 11 years) (p < 0.001). Of those patients with previously diagnosed CHF, 786 (70%) were registered according to the criteria listed in Table 1. These patients had the same age and gender characteristics as the whole study group. Echocardiography was performed in 38% of these patients (N = 295). More than half (55%) of them had left ventricular systolic dysfunction with an ejection fraction ≤45% (33% women, mean age 78 years, SD10 years compared to 67% men, mean age 75 years, SD 10 years) (p = 0.09); 18% had heart failure with preserved ejection fraction (HFPEF) (64% women, mean age 79 years, SD 8 years compared to 36% men, mean age 76 years, SD 9 years) (p = 0.20); and 16% had CHF that was due to valvular disease (53% women, mean age 84 years, SD 9 years compared to 47% men, mean age 77 years, SD 11 years) (p = 0.003). 11% of the echocardiography examinations were difficult to classify due to several potential causes of CHF. Of the 786 CHF patients with deterioration, only 2% (N = 15) could be sent home directly from the ED after acute medical treatment while 4% (N = 31) were included in a randomised trial assessing the feasibility of home care in this context [27]. The rest of the patients were admitted to hospital because of serious conditions (e.g., pneumonia/respiratory disease, myocardial infarction, pulmonary oedema, anaemia, need to monitor cardiac rhythm, pathological blood chemistry and communication difficulties resulting from dementia, stroke and other medical conditions) (Table 2).

**Table 2:** Reasons for hospital admission in patients with worsening CHF (The patients could be admitted for more than one reason)

Reason for hospital admission	Proportion	Number
Pneumonia/respiratory disease	35.4%	278

Need to monitor cardiac rhythm	15.6%	123

Communication problem (such as dementia, stroke and aphasia)	22.3%	175

Pulmonary oedema	11.3%	89

Myocardial infarction	6.2%	49

Anaemia*	5.2%	41

Pathologic blood chemistry other than haemoglobin**	3.7%	29

Hypotension	2.1%	17

*S-Haemoglobinb < 100 g/L or a decrease of S-Haemoglobin > 20 g/L

** S-Creatinine > 250 μmol/L, S-Potassium > 5.5 mmol/L or < 3.4 mmol/L, S-Troponin T > 0.05 μg/L, Creatine kinase-MB > 5 μg/L, ASAT and ALAT > three times above the normal value

## Discussion

The most important finding in our study was that, of the 786 patients with worsening CHFseeking care in the ED, only 2% could be sent home directly while the rest were admitted to the hospital. In the patients with CHF, who underwent echocardiography 55% presented with systolic HF and 16% with valvular heart disease. The number of patients with valvular heart disease in the present study is higher than in previous studies [28,29]. Data on cause of CHF may vary substantially depending on the type of study. For instance, where community populations have been investigated [28] or populations of admitted HF patients [29], about 10% of the CHF patients had valvular disease. In clinical randomised trials these patients are usually excluded and thus the demographics between trials and practise differ in this context. The high prevalence of valvular disease in our study may have been due to the high age of the study population (mean age 79 years, SD 11 years). Despite patients' desire to go home directly after acute treatment from ED, they could not be sent home mainly because of other comorbidities. Pneumonia and other respiratory diseases were the most common reason for hospital admission among patients with CHF in our study. Other common reasons included the need for rhythm recording and communication problems (such as dementia, stroke and aphasia), which are in accordance with the pattern found in studies of patients readmitted for worsening CHF [24-26]. The major comorbidities reported in the literature in patients with CHF are CHD, cerebrovascular disease, COPD, diabetes mellitus, renal failure, and pneumonia [14]. Hence, even if the treatment of CHF has developed markedly in the past years, the reasons for admission of patients with worsening CHF remain unchanged. Furthermore, the signs and symptoms are similar over the previous 20 years for patients with worsening CHF who attend an ED [14]. The life-prolonging therapies offered today might postpone, but cannot significantly alter the presentation of worsening CHF We have previously shown that the prognostic importance of objective evidence of HF, e.g. left ventricular ejection fraction (LVEF) is increased with simultaneous presence of symptoms [30,31]. These findings suggest that self-assessed symptoms should serve as the starting point when planning treatment and care of these patients upon arrival at the ED. Symptoms are important for the interpretation and understanding of patients with CHF, as CHF is a syndrome characterized by a cluster of symptoms. Symptoms are the foundation for how we classify disease and symptoms can only be experienced and described by the one who is affected, i.e. patients. Therefore the patient's own experience of illness must be taken seriously irrespective of type of care [32]. Research indicates that home-based HF care after initial discharge from hospital reduces rehospitalisation and mortality [23]. Many patients treated for CHF experience worsening symptoms long before seeking medical attention [22], symptoms that might have been possible to manage at home rather than in hospital if discovered earlier. Considerable effort has been devoted to educate patients and their relatives in identifying symptoms and signs of worsening CHF but none of these models seem to be sufficient as suggested by the fact that when CHF patients seek the ED they need to be admitted because of different complications. Our findings indicate that communication about symptoms between the health professionals and the patient is most important. In this context person-centeredness (partnership with and empowerment of the patient and family), should be integrated in CHF management programs [21]. Meanwhile, it seems that once the condition of patients with CHD has deteriorated to the extent that they must seek emergency care, hospital admission with rapid stabilisation is the most effective approach to improving care of patients with CHF mainly because of comorbidities from other major diseases.

## Limitations

The present study is small and single-centred and therefore the results cannot be generalised beyond the context of the study. However, the pattern of patients in our study corresponds to other studies in the same area [24-26]. Another limitation is that the results from the echocardiogram were available for only 38% of the eligible patients. This low figure makes the findings of a high percentage of valvular heart disease among the patients who required hospital admission somewhat uncertain.

## Conclusion

In spite of our knowledge that the patients with CHF delay in seeking care while worsening, they are in rapid need of help once they decide to seek care at the ED. Patients with severe chronic illness like CHF with symptomatic deterioration should be attended with focus on symptom relief and without additional emergency room investigations before hospital admission. A person-centered approach of care will facilitate both the patient and health care system. It is of utmost importance to listen to the patient history on symptom deterioration instead of solely focus on objective findings to provide rapid and adequate care.

## Competing interests

The authors declare that they have no competing interests.

## Authors' contributions

MS Participated in design of study, collected the data, performed statistical analysis and drafted the manuscript, HP collected the data, IE participated in design of study, KS participated in design of study, MS participated in design of study and helped to draft of manuscript. All authors read and approved the final manuscript.
